# High Pressure Processing Impact on Alternariol and Aflatoxins of Grape Juice and Fruit Juice-Milk Based Beverages

**DOI:** 10.3390/molecules26123769

**Published:** 2021-06-21

**Authors:** Noelia Pallarés, Albert Sebastià, Vicente Martínez-Lucas, Mario González-Angulo, Francisco J. Barba, Houda Berrada, Emilia Ferrer

**Affiliations:** 1 Preventive Medicine and Public Health, Food Science, Toxicology and Forensic Medicine Department, Faculty of Pharmacy, Universitat de València, Avda. Vicent Andrés Estellés, Burjassot, 46100 València, Spain; noelia.pallares@uv.es (N.P.); alsedu@alumni.uv.es (A.S.); marluvi@alumni.uv.es (V.M.-L.); emilia.ferrer@uv.es (E.F.); 2 Hiperbaric, S.A., C/Condado de Treviño, 6, 09001 Burgos, Spain; m.gonzalez@hiperbaric.com

**Keywords:** aflatoxin B1, alternariol, high-pressure processing, juice models, dispersive liquid–liquid microextraction, liquid chromatography coupled to tandem mass spectrometry

## Abstract

High-pressure processing (HPP) has emerged over the last 2 decades as a good alternative to traditional thermal treatment for food safety and shelf-life extension, supplying foods with similar characteristics to those of fresh products. Currently, HPP has also been proposed as a useful tool to reduce food contaminants, such as pesticides and mycotoxins. The aim of the present study is to explore the effect of HPP technology at 600 MPa during 5 min at room temperature on alternariol (AOH) and aflatoxin B1 (AFB1) mycotoxins reduction in different juice models. The effect of HPP has also been compared with a thermal treatment performed at 90 °C during 21 s. For this, different juice models, orange juice/milk beverage, strawberry juice/milk beverage and grape juice, were prepared and spiked individually with AOH and AFB1 at a concentration of 100 µg/L. After HPP and thermal treatments, mycotoxins were extracted from treated samples and controls by dispersive liquid–liquid microextraction (DLLME) and determined by HPLC-MS/MS-IT. The results obtained revealed reduction percentages up to 24% for AFB1 and 37% for AOH. Comparing between different juice models, significant differences were observed for AFB1 residues in orange juice/milk versus strawberry juice/milk beverages after HPP treatment. Moreover, HPP resulted as more effective than thermal treatment, being an effective tool to incorporate to food industry in order to reach mycotoxins reductions.

## 1. Introduction

Consumers’ demand for fresh fruits and vegetables has increased over the last years due to healthy lifestyle recommendations. A diet rich in fresh fruits and vegetables is related with a lower risk of developing cardiovascular, cancer, chronic conditions, cataracts, asthma, and bronchitis diseases. Most of these beneficial effects are attributed to the high content of micronutrients and bioactive compounds available in fruits and vegetables, such as vitamins, minerals, phenolic compounds, carotenoids, etc. [1,2]. Fruits and vegetable juices are highly popular beverages amongst consumers, seen as a healthy and easy option to meet the goals of five daily serving of fruits and vegetables [3]. However, juices made from concentrates or purees have recently come under fire regarding calorie and sugar concerns. Moreover, pasteurization used as standard method to extend juice products shelf-life causes some disadvantages such as change of color and loss of some nutritional and aromatic compounds [4,5]. Minimal processing nonthermal techniques, such as ultraviolet light (UV), pulsed electric fields (PEF), ultrasounds (US) and high-pressure processing (HPP), have emerged in the last years as good alternative options to keep these products fresh [6]. These techniques are being applied in the food industry to supply freshly squeezed juices and smoothies [7,8,9,10].

HPP is an environmentally friendly technique that is employed in food preservation to allow the inactivation of pathogenic microorganisms and vegetative spoilage without a negative impact on taste, texture, appearance, and nutritional value. This technology is governed by the Le Chatelier’s principle [11]. Pressure is transmitted uniformly and instantaneously throughout the food system independently of the size and geometry of the food. The temperature, pressure and exposure time are the processing parameters that characterize the treatment. In food preservation, the pressures applied ranged between 100 and 600 MPa, using mild temperatures (4–20 °C) and treatment period between several seconds to minutes [12]. Under HPP treatment, low molecular weight molecules, such as vitamins, minerals and aroma compounds, are rarely affected due the low compressibility of covalent bounds, while it can tune non-covalent interactions like hydrogen bonds and destabilize the hydrophobic effect, modifying the tertiary and quaternary spatial structure of macromolecules such as proteins and starch [13]. This technology satisfies consumers’ demand for fresh-like products, obtaining foods with similar characteristics to those of fresh products. Some of the HPP current commercial applications are mainly focused on food safety and shelf-life extension, as well as nutritional, organoleptic and sensorial quality. Moreover, HPP has also a great potential to promote the recovery of health-related compounds, improving health food attributes increasing the bioavailability of micronutrients and phytochemicals, reducing the allergenic potential of some foods, preserving healthy lipids, reducing salt intake by increasing the saltiness perception and reducing food processing contaminants formation [14].

More recently, HPP has also been proposed as a useful tool for removing foods contaminants, such as pesticides and mycotoxins. The process efficiency depends on several factors such as the processing parameters, the chemical structure of the pesticide/mycotoxin, and the food matrix [15]. Some studies have reported significant decreases in mycotoxins and pesticides after HPP treatment; however, the number of compounds studied is limited and more data is necessary for a better understanding of the role of this technology in pesticides and mycotoxins degradation, and to establish the optimal conditions [16,17,18,19].

Mycotoxins are toxic natural contaminants of food and feeds that are produced by various fungi from *Aspergillus*, *Alternaria*, *Fusarium*, and *Penicillium* generas. The contamination can occur during pre-harvest when the crop plant is growing or in post-harvest processing, packaging, distribution, and storage steps. Poor agricultural, harvesting and manufacturing practices can promote fungal growth and consequently the risk of mycotoxin production. In general, mycotoxins are chemically and thermally stable during food processing [20,21]. Concerning to the most relevant mycotoxins reported in food, aflatoxins (AFs) are produced by *Aspergillus* species; ochratoxin A (OTA) by both *Aspergillus* and *Penicillium* generas; trichothecenes, zearalenone (ZEA), fumonisins (FBs) and emerging mycotoxins are produced by *Fusarium* species; and finally *Alternaria* species are responsible of altenuene (ALT), alternariol (AOH), alternariol methyl ether (AME), altertoxin (ATX), and tenuazonic acid (TA) production (Table 1) [22]. Mycotoxins acute and chronic dietary exposures is related with a variety of adverse health effects in humans and animals. The long-term exposure to high doses is linked to mutagenicity, carcinogenicity, teratogenicity, hepatotoxicity, nephrotoxicity, gastrointestinal toxicity, immunotoxicity and neurotoxicity. Mycotoxin occurrence has been widely reported in cereals, dried fruits, spices, coffee, fruits and their by-products. In fruits and their processed products, Patulin (PAT), AFs, OTA and the *Alternaria* toxins such as AOH, AME and ALT constitute the most common mycotoxins [23].

AFs are the most important mycotoxins owing to their genotoxic carcinogenic properties, being AFB1 among the most potent mutagenic and carcinogenic substances known. AFs toxicity must be distinguished between acute and chronic, chronic toxicity being the most common form of aflatoxicosis [24]. Chronic exposure induces liver cancer and adverse effects in reproductive and immune systems. AFs are very stable compounds, that may resist several food processing operations, thus this AFs can be a problem in processed foods [22]. Among *Alternaria* toxins, AOH is the most predominantly occurring in food and is related with mutagenicity and genotoxicity [25]. Both AFB1 and AOH have been reported by several authors in different types of juices [26,27,28,29].

To the best of our knowledge, the information available in literature about the effect of HPP technique on mycotoxins reduction in juices matrices is limited. In this sense, HPP technology has been explored in fruit juices for PAT and AFs removal, achieving reduction percentages up to 51% [30,31,32].

Therefore, the main aim of the current study is to explore the effect of HPP technology on AOH and AFB1 reduction in different juice models and to compare it with the effect of thermal treatment.

## 2. Results and Discussion

### 2.1. Effect of HPP on AFB1 and AOH Reduction

**Figure 1:**
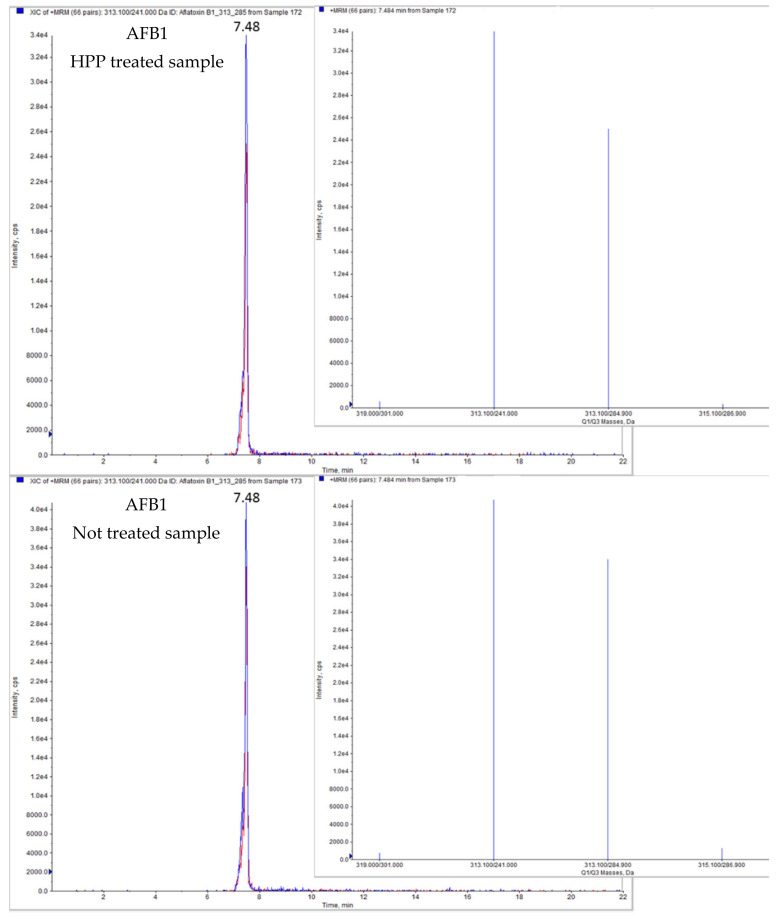
LC-MS/MS-IT chromatogram of grape juice sample contaminated by AFB1 treated by HPP vs. non-treated.

The different juice models were prepared and spiked with AFB1 or AOH at concentrations of 100 µg/L, subsequently different aliquots were separated to be employed as not treated controls, and the rest of the samples were subjected to HPP or thermal treatment, respectively. Comparing mycotoxins contents in HPP treated samples with their respective controls, the results obtained revealed a significant reduction after the application of HPP treatment. Levels quantified in treated samples ranged from 76.29 µg/L to 92.92 µg/L for AFB1, and from 62.85 µg/L to 72.27 µg/L for AOH, corresponding to reduction percentages up to 24% for AFB1 and 37% for AOH. Figure 1 shows the chromatograms of grape juice sample spiked with AFB1, comparing HPP-treated vs. no treated sample.

Concerning the information available in the literature regarding the use of HPP technology to decrease mycotoxins, there is a lack of details about the impact of HPP on AFB1 and AOH contents, being the effect on PAT more widely studied in juice matrices. In this sense, similarly to the present work, Hao et al. [31] obtained reduction percentages up to 31% after investigating the effect of HPP treatment under conditions of 600 MPa during 300 s at 11 °C in different juice models spiked with PAT at a concentration of 200 µg/L. The different apple-based juice models studied by these authors consisted of apple and spinach; pineapple, apple and mint; apple, carrot, beet, lemon and ginger; and romaine, celery, cucumber, apple, spinach, kale parsley and lemon, the last one being the juice model where the higher reduction percentage was observed (31%). Slightly higher decrease percentages were obtained by Avsaroglu et al. [30], who reported PAT reduction up to 51.16% by HPP treatment (at 400 MPa and 30 °C) and to 62.11% under pulsed-high hydrostatic pressure p-HPP (6 pulse × 50 s, 300 MPa and 50 °C) in apple juice spiked at 5, 50 and 100 ppb. These authors studied different pressure treatments (300–500 MPa) in combination with different temperatures (30–50 °C) and pulses (6 pulses × 50 s and 2 pulses × 150 s). Comparing both treatments, HPP resulted as more effective at high concentrations, while p-HPP was more efficient at low concentrations.

Regarding AFs, similar reduction percentages for AFB1 (17%), AFB2 (14%), AFG1 (19%) and AFG2 (29%) were obtained in a previous work after evaluating the impact of HPP (500 MPa/5 min) in a processed commercially grape juice spiked with AFs at the same concentration (100 µg/L) [32].

Concerning *Alternaria* mycotoxins (AOH and AME), Ioi [33] studied the effect of thermal processing (90 or 121 °C for 10 or 20 min) and HPP treatment (300 or 600 MPa for 3 or 5 min) on AOH and AME spiked at concentrations of 80, 200, and 500 µg/kg in fresh tomato juices. Thermal treatment resulted in reductions of AOH ranging from 0.9% to 14.5% and AME from 1.0% to 15.3%, while HPP treatment produced reduction percentages in a range comprised between 5.8% and 25.3% for AOH and between 1.7% and 12.9% for AME. In the present study, slightly higher reduction percentages were achieved for AOH in the different model juices studied (28–37%) after HPP treatment at 600 MPa during 300s.

In other food matrixes, such as black olives and maize, HPP has also been reported as an effective tool for mycotoxins reduction. For instance, Tokusoglu et al. [34] studied the effect of HPP at 250 MPa during 5 min on citrinin reduction in black table olives spiked at 1, 2.5, 10, 25 and 100 ppb. While 1 ppb of CIT contamination was absolutely inhibited as 100%, only 1.3% of reduction was achieved by these authors at 100 ppb. In other study Kalagatur et al. [18] obtained Deoxynivalenol (DON) and ZEA reduction rates up to 100% in maize treated by HPP at 550 MPa and 45 °C during 20 min.

### 2.2. Comparison of the Results Obtained after Applying HPP in the Different Juice Models Studied

Per type of juice model studied, AFB1 contents obtained after HPP treatment were 76.29 ± 8.17 µg/L, 92.92 ± 8.66 µg/L and 87.4 ± 6.25 µg/L in orange juice/milk beverage, strawberry juice/milk beverage and grape juice, respectively, corresponding to reduction percentages of 24, 7 and 13%, respectively (Table 2). Orange juice/milk beverage was the juice model where a higher AFB1 reduction was achieved. Statistically significant differences were observed between orange juice/milk and strawberry juice/milk beverages (*p* < 0.05), revealing a possible effect of matrix on results obtained. As it has been reported by other authors, the degradations obtained could be dependent of juice model constituents. For instance, Hao et al. [31] evidenced that PAT degradation assisted by HPP was dependent on juice constituents. These authors observed that the reduction obtained was a result of pressure in combination with reaction of patulin lactone rings with reactive groups contained in juices such as thiol group. This idea was supported by the fact that among all juice models studied, romaine, celery, cucumber, apple, spinach, kale parsley and lemon juice, in which the highest level of thiol group was presented, the higher reduction of PAT was achieved. In another study, the differences in the results obtained after HPP treatment in apple juices were also attributed to the action of sulphhydryl group, such as glutathione or cysteine, bearing PAT molecules [30]. In a previous study, higher emerging mycotoxin (ENNs and BEA) reductions were observed in smoothie samples (from 56 to 70%) compared to grape juice samples (from 43 to 53%) under pulsed electric field treatment (PEF) of 3 kV/cm and specific energy of 500 kJ/kg. This might be attributed to the fact that smoothies constitute a more complex matrix [35].

Thus, HPP technology may assist the reaction of mycotoxins with different reactive groups present in food matrix. This idea was verified by Merkulow and Ludwig [36], who studied the adduct formation of patulin with cysteine groups promoted by high hydrostatic pressure and observed that reaction resulted in different adducts built, being dependent on the environmental conditions. Other researchers, Rodríguez-Bencomo et al. [37], observed the formation of patulin–glutathione conjugates as result of other emerging technology application (pulsed light). Moreover, although HPP technology does not impact on covalent bounds, can affect ionic and hydrogen bonding, changing the native structure of macromolecules, such as proteins and starch, exposing reactive groups compounds in juice environment and consequently resulting in mycotoxins mitigation. However, more information is necessary for a better understanding of the mechanism of action involved.

Regarding AOH, contents of 71.05 ± 10.61 µg/L, 72.27 ± 12.26 µg/L and 62.85 ± 8.97 µg/L were quantified in orange juice/milk beverage, strawberry juice/milk beverage and grape juice, respectively, after HPP treatments, resulting in reduction percentages of 29, 28 and 37%, respectively (Table 2). In this case, no statistically significant differences were observed (*p* > 0.05) among the different juice models studied.

### 2.3. Effect of Thermal Treatment on AFB1 and AOH Reduction

The possible action of thermal treatment at 90 °C during 21 s was also studied in order to compare the effects of HPP and thermal treatment in the reduction of AFB1 and AOH contents. Heating at 65–95 °C during several seconds to various minutes is a normal practice applied in commercial juices preservation [38]. In this sense, no AFB1 reduction was achieved after thermal treatment in orange juice/milk beverage and in strawberry juice/milk beverage observing concentrations near to 100 µg/L, while in grape juice 88.37 µg/L were obtained, corresponding to a reduction percentage of 12% (Table 2). Comparing the results obtained in both treatments only statistically significative differences (*p* < 0.05) were obtained comparing AFB1 contents obtained after HPP and thermal treatments in orange juice/milk beverage.

Concerning AOH, only 7% reduction was observed after thermal treatment in grape juice with quantified amount of 92.60 µg/L, but no reductions were observed in orange juice/milk beverage and strawberry juice/milk beverage (Table 2). Thus, statistically significative differences (*p* < 0.05) were observed comparing the effect of HPP vs. thermal treatment on AOH contents in all juice models studied. These results suggest that HPP can be a most effective tool in AFB1 and AOH removal from juice matrixes than the conventional thermal pasteurization process. Similar to the present study, Ioi [33] also reported HPP as a more effective technique in AOH and AME reduction in tomato fresh juices than high temperature processing.

In general, most mycotoxins are known to be heat-resistant at temperatures of conventional food processing (80–121 °C), thus little or no reduction is expected to occur during cooking processes such as boiling and frying, or pasteurization. Several factors such as the initial mycotoxins levels, the type of mycotoxin, the temperature, the processing time, the degree of heat penetration, the Ph and the ionic strength of food, among others are related with the level of mycotoxins degradation recorded [39]. Regarding to the information available in literature about the effect of thermal treatment of pasteurization on AFs and AOH contents, Elhariry et al. [40] also did not report visible changes in *Alternaria* toxins after the pasteurization of pomegranate juice. In milk, Govaris et al. [41] not observed significant differences in the AFM1 contents spiked in milk at 0.050 and 0.10 g/L after heating at 92 °C during 3 min, suggesting that AFM1 is relatively stabled during pasteurization and sterilization processes. In contrast, Deveci [42] achieved AFM1 reductions between 12 and 9% in artificially contaminated milk (1.5 μg/L and 3.5 μg/L) after pasteurization treatment under 72 °C for 2 min.

Higher processing temperatures (approximately 150 °C) achieved during roasting or extrusion industrial processes resulted in higher AOH and AFs reduction percentages (17–90%) in function of temperature and time [43,44].

## 3. Materials and Methods

### 3.1. Reagents and Chemicals

Acetonitrile (ACN), methanol (MeOH) (HPLC grade) and chloroform (CHCl_3_) solvents (99% grade) were supplied by Merck (Darmstadt, Germany). Ethyl acetate (EtOAc) (HPLC grade 99.5+ %) was purchased from Alfa Aesar (Karlsruhe, Germany). The deionized water with resistivity >18 MΩ cm^−1^ employed for prepare mobile phase was obtained using a Milli-Q SP^®^ Reagent Water System (Millipore Corporation, Bedford, MA, USA). Prior to the use, all solvents necessary to prepare mobile phases were filtered through a 0.45-μm cellulose filter supplied by Scharlau (Barcelona, Spain). Sodium chloride (NaCl) was purchased from VWR Chemicals (Leuven, Belgium), ammonium formate (99%) was obtained from Panreac Quimica S.A.U. (Barcelona, Spain) and formic acid (reagent grade ≥ 95%) was acquired from Sigma-Aldrich (St. Louis, MO, USA). Prior to injection, all samples were filtered through a 13 mm/0.22 μm nylon filter supplied by Membrane Solutions (TE, USA). AFB1 and AOH mycotoxins standards were supplied by Sigma (St. Louis, MO, USA) and were prepared in methanol at concentration of 1000 mg/L. Then, the appropriate working solutions were prepared from the stock solutions. All solutions were stored at −20 °C until the analysis.

### 3.2. Samples

Different juice models were prepared: orange juice/milk beverage, strawberry juice/milk beverage and grape juice. The composition of the juices is detailed in Table 3.

In order to prepare the different juice models, the water was heated at 50 °C and then pectin and sugar, previously crushed, were added under agitation. Subsequently, the milk was heated to 50 °C prior to adding to the mixture with constant agitation. Then, the mixture was cooled, and the corresponding juice and citric acid were added and kept under stirred until the sample was homogeneous. To prepare the grape juice model, milk was not employed. A total volume of 1.5 L was prepared for each juice model, and aliquots were taken to test the absence of mycotoxins. Subsequently, a volume of 1.2 L was spiked individually by AFB1 and AOH at a concentration of 100 μg/L. Finally, aliquots were taken in triplicate to be employed as not-treated controls, and the rest of the juice was bottled per triplicated in a 330 mL bottles and refrigerated at 4 °C.

### 3.3. HPP Procesing Treatment

A high-pressure equipment Hiperbaric 55 (Burgos, Spain), capable of producing a maximum working pressure of 600 MPa, was employed. The instrument was equipped with 55 L pressure chamber filled with water at temperature between 10 and 12 °C as a pressure-transferring medium. Temperature increased in samples during pressurization process due to the adiabatic heat at approximately 3 °C = 100 MPa. The come-up rate was approximately 3.6 MPa/s.

The plastic bottles containing the different juice models were placed in plastic bags that were filled with a solution of hydrogen peroxide and deionized water in the proportion 1/25, respectively. Each bag contained the solution and the three replicates for each mycotoxin and type of juice model. In addition, a second bag was settled, covering the first one, then the vacuum was applied, and the bag was heat sealed. Juice samples were treated under pressure of 600 MPa during treatment time of 5 min.

### 3.4. Thermal Treatment

Thermal treatment (HT) was conducted in a Julabo circulating water bath (Seelbach, Germany) where temperature was set at 90 °C. According to Barba [45], the treatment performed to simulate conventional pasteurization consisted of applying 90 °C during 21 s. After treatment, all samples were immediately cooled in ice-water and then stored under refrigeration at 4 °C until the analysis. All the experiments were carried out in triplicate. Three aliquot portions of the samples were not heat treated and separated to be employed as not treated controls.

### 3.5. Dispersive Liquid-Liquid Microextraction Procedure (DLLME)

Mycotoxins were extracted from treated samples and controls using DLLME procedure and the method was previously validated in a previous work [27]. For this, 5 mL of juice beverage were placed in 10 mL conical tub with 1 g of NaCl and shaken for one minute. In a first step, a mixture of dispersant solvent (950 μL of AcN) and extractant solvent (620 μL of EtOAc) was added, then was shaken for one minute more, resulting in a cloudy solution of the three components. After centrifugation at 4000 rpm during 5 min, the phases were separated. The organic phase, located at the top of the tub, was separated and placed into other conical tube. Then, in a second step, the mixture of dispersant solvent (950 μL of MeOH) and extractant solvent (620 μL of CHCL_3_) was added to the remaining residue. After shake and centrifugate again, the organic phase located at the bottom of the tube was separated and placed with the first organic phase recovered. Both organic phases were evaporated in a Turvovoap LV Evaporator (Zymark, Hoptikinton, MA, USA) employing a nitrogen stream using a Turvovap and finally were reconstituted in a vial employing 1 mL of 20 mM ammonium formate (MeOH/ACN) (50/50 *v*/*v*) and filtered through a 13 mm/0.22 μm nylon filter prior to the injection in LC-MS/MS-IT system.

### 3.6. LC-MS/MS-IT Determination

The determination was carried on in an Agilent 1200 chromatograph (Agilent Technologies, Palo Alto, CA, USA) equipped with 3200 QTRAP^®^ (Applied Biosystems, AB Sciex, Foster City, CA, USA) with Turbo Ion Spray (ESI) electrospray ionization. The QTRAP analyzer combines a fully functional triple quadrupole and a linear ion trap mass spectrometer. A Gemini-NX column C18 (Phenomenex, 150 mm × 4.6 mm, 5 particle size) preceded by a guard column was employed to perform the chromatographic separation. The mobile phases employed consisted in 5 mM ammonium formate, 0.1% formic acid water and 5 mM ammonium formate, 0.1% formic acid methanol. The gradient program started with a proportion of 0% for eluent B; increasing to 100% in 10 min, then decreasing to 80% in 5 min, and finally to 70% in 2 min. In the next 6 min, the column was cleaned, readjusted to initial conditions, and equilibrated during 7 min. The parameters were fixed as follows: flow rate set at 0.25 mL/min, injection volume of 20 µL and oven temperature was at 40 °C.

For the analysis, the Turbo Ion Spray operated in positive ionization mode (ESI+) and nitrogen was served as nebulizer and collision gas. During the analysis, ion spray voltage was set at 5500 V, the curtain gas was set at 20 (arbitrary units), the nebulizer (GS1) and TIS (GS2) gases at 50 and 50 psi, respectively, and the probe temperature (TEM) was fixed at 450 °C.

### 3.7. Method Validation

The methodology proposed was previously validated in a previous work [27]. For this, recovery, repeatability (intraday precision), reproducibility (interday precision), matrix effects, and limits of detection (LOD) and quantification (LOQ) parameters were characterized according to the Commission Decision [46]. Recovery experiments were performed at three contamination levels (50, 100, 200 µg/L), the values obtained ranged from 69 to 83% for AFB1 and from 90 to 114% for AOH. The Intra-day and inter-day precision were lower than 14% and 19%, respectively. Regarding matrix effect experiments, the signal suppression-enhancement observed was 80% for AFB1 and 67% for AOH. The LODs and LOQs observed were 0.3 and 1 µg/L, respectively, for both AFB1 and AOH. Finally, a good linearity was obtained with regression coefficients higher than 0.990.

### 3.8. Statistical Analyses

The results obtained were analyzed employing an analysis of variance (3way ANOVA) followed by Tukey’s test to determine the significance of differences between treatments and juice models and mycotoxins concentrations. A probability value of *p* < 0.05 was considered significant. All statistical analyses were performed employing the software GraphPad Prism8.0.2 (GraphPad Software, San Diego, CA, USA). Values obtained were expressed as mean± standard deviation (SD). All analysis performed were applied in triplicate.

## 4. Conclusions

HPP treatment is presented here as an effective tool to incorporate to food industry in order to decrease mycotoxins contents with a minimum impact on quality attributes.

Reductions percentages up to 24% (AFB1) and 37% (AOH) have been obtained in the different juice models studied after HPP treatment at 600 MPa during 5 min. Significant differences were obtained comparing AFB1 contents between orange juice/milk beverage and strawberry juice/milk, while no significant differences were observed for AOH. Thermal treatment did not reach any mycotoxins reduction in orange juice nor strawberry juice, and only 12% of decreasing for AFB1 and 7% for AOH in grape juice. Significant differences were obtained comparing AOH contents after HPP and thermal treatments, being HPP technology more effective in mycotoxins removal than pasteurization thermal treatment. Future investigation should be carried out to provide new insights into the effect of HPP technology in mycotoxins reduction, as well as to explore the potential of combining HPP with other alternative treatments, such as ultrasound or pulsed electric fields.

## Figures and Tables

**Table 1:** Most relevant mycotoxins and their species producers.

Mycotoxin	Molecular Structure	Species Producers
AFLATOXINS AFs (AFB1, AFB2, AFG1, AFG2)	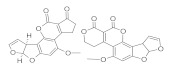	*Aspergillus section Flavi*
OCHRATOXIN A	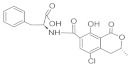	*Aspergillus section Nigri* *Aspergillus section Circumdati* *Penicillium verrucosum* *Penicillim nordicum*
FUMONISINS (FB1, FB2, FB3)	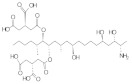	*Fusarium section Liseola*
ZEARALENONE (ZEA)	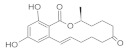	*Fusarium graminearum (F. roseum), F. culmorum, F. equiseti, F. cerealis, F. verticillioides, F. incarnatum*
TRICHOTHECENES	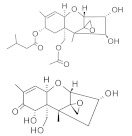	
A: HT-2, T-2		*Fusarium acuminatum, F. poae, F. sporotrichioides, F. langsethiae*
B: Deoxynivalenol (DON), 3DON, 15DON, Nivalenol (NIV)		*Fusarium graminearum, F. culmorum, F. cerealis*
FUSARIUM EMERGING MYCOTOXINS	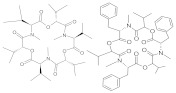	
Enniatins (ENNs)		*Fusarium avenaceum, F. tricinctum*
Beauvericin (BEA)		*Fusarium avenaceum, F. sporotrichioides, F. poae,* *F. langsethiae, Fusarium section Liseola*
ALTERNARIA MYCOTOXINS	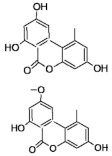	
Alternariol (AOH)		*Alternaria alternata*
Alternariol monomethyl ether (AME)		*Alternaria alternata, A. solani*
Tenuazonic acid (TeA)		*Alternaria alternata*
Altertoxins (ALTs)		*A. tenuissima*
Altenuene (ALT)		*Alternaria alternata*

**Table 2:** Contents of AFB1 and AOH obtained after high-pressure processing (HPP) and thermal treatment in different juice models samples spiked at 100 µg/L.

Mycotoxin	Contents (µg/L) after HPP Treatment			Contents (µg/L) after Thermal Treatment		
	Orange Juice/Milk Beverage	Strawberry Juice/Milk Beverage	Grape Juice	Orange Juice/Milk Beverage	Strawberry Juice/Milk Beverage	Grape Juice
AFB1	76.29 ± 8 ^A,^*	92.92 ± 9 ^B^	87.40 ± 6	100.00 ± 4	100.00 ± 4	88.37 ± 9
AOH	71.05 ± 11 *	72.27 ± 12 *	62.85 ± 9 *	100.00 ± 3	100.00 ± 12	92.60 ± 12

Note: different letters (A, B) between different juice models show significant differences (*p* < 0.05) between juice types per each mycotoxin. * indicates that contents obtained after HPP are significantly different (*p* < 0.05) from those obtained by thermal treatment.

**Table 3:** Amounts for the ingredients employed (per 100 mL) to prepare the different juice models.

Ingredients	Orange Juice/Milk Juice	Strawberry Juice/Milk Juice	Grape Juice
Juice	50 mL	30 mL	60 mL
Skim milk	20 mL	20 mL	0 mL
Bottled water	30 mL	50 mL	40 mL
Pectin	0.3 g	0.3 g	0.3 g
Sugar	7.5 g	7.5 g	7.5 g
Citric acid	0.1 g	0.1 g	0.1 g

## Data Availability

Not applicable.

## Sample Availability

Samples of the compounds are available from the authors.
